# Adaptor protein APPL1 links neuronal activity to chromatin remodeling in cultured hippocampal neurons

**DOI:** 10.1093/jmcb/mjaa058

**Published:** 2020-10-26

**Authors:** Yu Wu, Xinyou Lv, Haiting Wang, Kai Qian, Jinjun Ding, Jiejie Wang, Shushan Hua, Tiancheng Sun, Yiting Zhou, Lina Yu, Shuang Qiu

**Affiliations:** 1 Department of Neurobiology, Second Affiliated Hospital, Zhejiang University School of Medicine, Hangzhou 310058, China; 2 Department of Anesthesiology, Second Affiliated Hospital, Zhejiang University School of Medicine, Hangzhou 310058, China; 3 Department of Biochemistry, Second Affiliated Hospital, Zhejiang University School of Medicine, Hangzhou 310058, China; 4 Department of Orthopaedic Surgery, Second Affiliated Hospital, Zhejiang University School of Medicine, Hangzhou 310058, China; 5 NHC and CAMS Key Laboratory of Medical Neurobiology, MOE Frontier Science Center for Brain Research and Brain-Machine Integration, School of Brain Science and Brain Medicine, Zhejiang University, Hangzhou 310058, China

**Keywords:** APPL1, excitation‒transcription coupling, synaptic plasticity, chromatin remodeling, gene transcription

## Abstract

Local signaling events at synapses or axon terminals are communicated to the nucleus to elicit transcriptional responses, and thereby translate information about the external environment into internal neuronal representations. This retrograde signaling is critical to dendritic growth, synapse development, and neuronal plasticity. Here, we demonstrate that neuronal activity induces retrograde translocation and nuclear accumulation of endosomal adaptor APPL1. Disrupting the interaction of APPL1 with Importin α1 abolishes nuclear accumulation of APPL1, which in turn decreases the levels of histone acetylation. We further demonstrate that retrograde translocation of APPL1 is required for the regulation of gene transcription and then maintenance of hippocampal late-phase long-term potentiation. Thus, these results illustrate an APPL1-mediated pathway that contributes to the modulation of synaptic plasticity via coupling neuronal activity with chromatin remodeling.

## Introduction

Activity-dependent regulation of gene expression (excitation‒transcription coupling) is a powerful means by which neurons build up stable changes of neuronal properties, a process that is essential for long-term synaptic plasticity and memory (Impey et al., 1996; West et al., 2002; West and Greenberg, 2011). A great number of studies in these years has been focused on identifying pathways that couple synapse to the nucleus to elicit transcriptional responses (Ch’ng et al., 2012; Bading, 2013; Karpova et al., 2013; Ma et al., 2014).

Calcium signals are the major route for communication of synaptic activity to the nucleus (Adams and Dudek, 2005; Bading, 2013). Synaptic activity induces a rapid and transient rise in calcium levels within the postsynaptic specialization, which then triggers the release of internal calcium stores from the endoplasmic reticulum, creating a regenerative calcium wave that propagates toward the soma (Dolmetsch et al., 2001; Thiagarajan et al., 2005; Zhao et al., 2005; Adams et al., 2009). The influx of calcium at the soma or the nucleus acts as a second messenger to initiate a cascade of signaling events and results in the activation of a program of gene expression within minutes. The active transport of signaling molecules is another well-known mechanism for synapse-to-nucleus signal coupling. In recent years, a burgeoning list of signaling molecules has been identified to be implicated in synapse to nucleus communication (Wellmann et al., 2001; Proepper et al., 2007; Dieterich et al., 2008; Lai et al., 2008; Jordan and Kreutz, 2009; Schmeisser et al., 2009; Marcora and Kennedy, 2010; Dubielecka et al., 2011; Ch’ng et al., 2012; VanLeeuwen et al., 2014). Most of these proteins directly associate with NMDA receptor (NMDAR) complex, making them in a privileged position to sense local synaptic events. Synaptic activity can drive the disassociation of these proteins from synapse, and then long-distance retrograde trafficking along microtubule via an association with motor proteins such as dynein (Liu et al., 2005; Ben-Yaakov et al., 2012; Karpova et al., 2013; Dent and Baas, 2014).

APPL1 and APPL2 are multifunctional adaptor proteins that contain an N-terminal bin1/amphiphysin/Rvs 167 domain, a pleckstrin homology domain, and a C-terminal phosphotyrosin binding (PTB) domain (Diggins and Webb, 2017). APPL1, as a Rab5 effector, can be recruited to a subset of Rab5-positive early endosomes and participate in vesicle trafficking (Miaczynska et al., 2004; Erdmann et al., 2007; Zoncu et al., 2009). Moreover, APPL1 facilitates cross-talk between different signaling pathways via its interaction with many receptors and signaling proteins through its PTB domain (Mao et al., 2006; Schenck et al., 2008; Cheng et al., 2012; Ryu et al., 2014; Galan-Davila et al., 2018). Furthermore, APPL1 plays additional role in the modification of gene expression in non-neuronal cells, mainly through directly shuttling into the nucleus to stimulates changes in chromatin remodeling (Miaczynska et al., 2004; Banach-Orlowska et al., 2009) or indirectly regulating the activity and nuclear location of other partners to promote transcription (Rashid et al., 2009; Banach-Orlowska et al., 2015).

APPL1 is also highly expressed in the central nervous system, although its function is far from well-known. Our previous work has shown that APPL1 couples synaptic NMDARs with downstream PI3K/AKT signaling pathway and participates in neuroprotective effect (Wang et al., 2012). In this study, we demonstrate that neuronal activity induces retrograde transport of APPL1 into the nucleus via the interaction of APPL1 with Importin α1. This dendritic APPL1-mediated pathway induces chromatin remodeling and thus regulates gene transcription and plays key roles in long-term synaptic plasticity.

## Results

### Neuronal activity induces nuclear accumulation of APPL1 in the cultured hippocampal neurons

**Figure 1:**
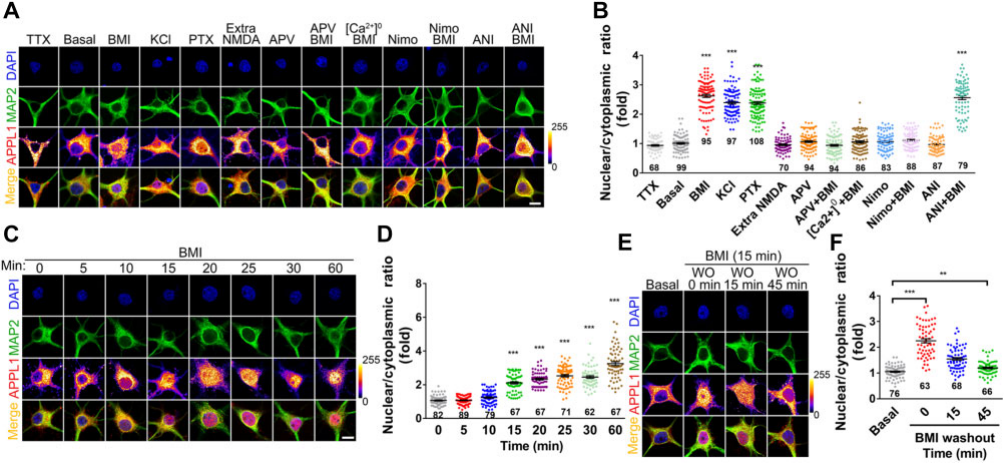
Synaptic activity induces nuclear accumulation of APPL1 in the cultured hippocampal neurons. (**A**) Cultured hippocampal neurons at DIV 14‒17 were untreated (basal) or treated with Tetrodotoxin (TTX, 1 μM), BMI/4-AP (BMI, 50 μM/2.5 mM), Picrotoxin (PTX, 50 μM), KCl (50 mM), extrasynaptic NMDAR activation protocol (Extra NMDA), APV (50 μM), Nimodipine (Nimo, 10 μM), or Anisomycin (ANI, 50 μM), respectively, for 1 h. Alternatively, hippocampal neurons were pretreated with APV, Nimodipine, calcium-free extracellular media ([Ca^2+^]^0^), or Anisomycin, respectively, for 30 min followed by treatment with BMI for another 1 h. After treatment, the cultures were immunostained with antibodies against MAP2 (green) and APPL1 (color lookup table, pixel intensities from 0 to 255) and with DAPI nuclear dye (blue). Scale bar, 10 μm. (**B**) Statistical analysis of the nuclear/cytoplasmic ratio of APPL1. (**C**) Hippocampal neurons at DIV 14‒17 were pretreated with Leptomycin B (10 nM) for 1 h, then treated with BMI for indicated times, and subsequently stained with antibodies against MAP2 (green) and APPL1 (color lookup table) and with DAPI nuclear dye (blue). Scale bar, 10 μm. (**D**) Statistical analysis of the nuclear/cytoplasmic ratio of APPL1. (**E**) Hippocampal neurons were treated with BMI for 15 min, washed out (WO), and then incubated in fresh media absent of BMI for different times as indicated. Neurons were stained with antibodies against MAP2 (green) and APPL1 (color lookup table) and with DAPI nuclear dye (blue). Scale bar, 10 μm. (**F**) Statistical analysis of the nuclear/cytoplasmic ratio. **P <* *0.01, ***P <* *0.005.

To examine whether APPL1 undergoes nuclear translocation in the cultured hippocampal neurons, we first used immunostaining with antibodies that specifically recognize APPL1 (Supplementary Figure S1A and B) to test the intracellular localization of endogenous APPL1 under basal conditions. APPL1 was present in several subcellular compartments, such as Golgi fractions (indicated by GM130), early endosomal membrane fractions (indicated by EEA1), and late endosomal membrane fractions (indicated by Rab7) (Supplementary Figure S1C). Moreover, APPL1 was wildly distributed in the soma and along the dendrites under basal conditions but was excluded from the nucleus of hippocampal neurons (Figure 1A).

Next, we incubated cultures with GABA_A_ receptor antagonist Bicuculline and K^+^ channel antagonist 4-aminopyridine (BMI/4-AP) together to drive excitatory synaptic transmission. As shown in Supplementary Figure S1D and E, the total amount of APPL1 was unchanged after incubation with BMI/4-AP for 1* *h. Interestingly, the distribution of APPL1 underwent a ∼2.5-fold increase in the nuclear/cytoplasmic intensity ratio (Figure 1A and B). Similar changes were evoked by incubation with another GABA_A_ receptor antagonist Picrotoxin (PTX) or depolarization with KCl (Figure 1A and B). In contrast, treatment with Na^+^ channels blocker Tetrodotoxin (TTX) to block action potentials or activation of extrasynaptic NMDARs showed no nuclear accumulation of APPL1 (Figure 1A and B). Furthermore, pretreatment with NMDAR antagonist APV or L-type voltage-gated calcium channels blocker Nimodipine (Nimo) or removal of calcium from the extracellular media completely abolished activity-induced nuclear accumulation of APPL1 (Figure 1A and B), indicating that the influx of extracellular calcium is essential for activity-induced nuclear APPL1 accumulation. To exclude the possibility that nuclear accumulation of APPL1 depends on protein synthesis, we pretreated cultures with Anisomycin, an inhibitor of protein synthesis and observed that treatment with Anisomycin did not affect activity-dependent nuclear accumulation of APPL1 (Figure 1A and B).

We also analysed the time course of nuclear APPL1 accumulation and observed that the nuclear/cytoplasmic intensity ratio of APPL1 was significantly increased when neurons were incubated with BMI/4-AP for 15* *min or longer, but not for 5 or 10* *min (Figure 1C and D). Similarly, we detected the nuclear and cytosolic levels of APPL1 using subcellular fractionation and observed that APPL1 was accumulated in the nucleus when neurons were incubated with BMI/4-AP for 40 min (Supplementary Figure S1F and G). Furthermore, when cultures were incubated with BMI/4-AP for 15 min followed by a quick washout, the immunoreactivity of APPL1 in the nucleus was gradually decreased (Figure 1E and F), indicating that nuclear accumulation of APPL1 is dynamically regulated by synaptic activity.

In contrast, we observed that APPL2, another isoform of APPL (Miaczynska et al., 2004), existed in the nucleus under basal conditions and the abundance of APPL2 in the nucleus showed no significant change after stimulation with BMI/4-AP for 1 h (Supplementary Figure S1H and I).

### APPL1 undergoes activity-dependent translocation along the dendrites

**Figure 2:**
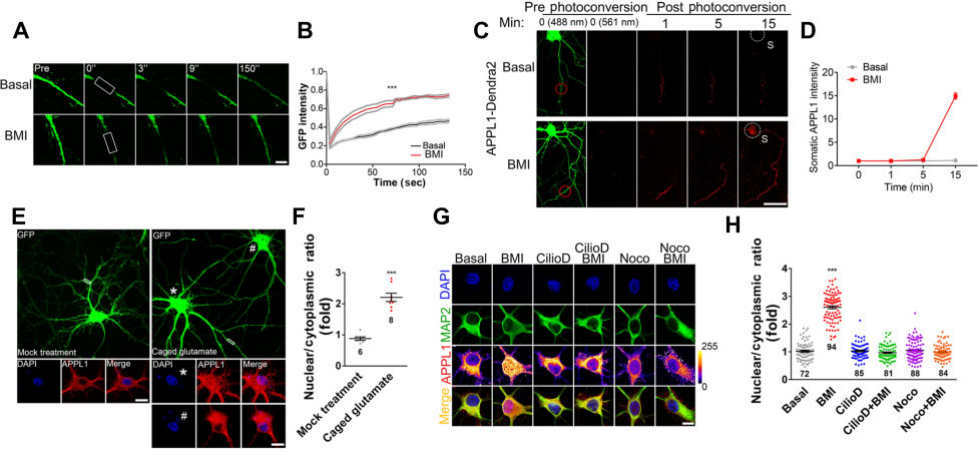
APPL1 undergoes neuronal activity-induced retrograde transport along the dendrites. (**A** and **B**) APPL1-GFP was transfected into hippocampal neurons at DIV 12 for 24 h before FRAP experiment. Neurons were incubated in Tyrode’s solution containing Torlox (10 nM) at 37°C and subsequently stimulated with BMI/4-AP (50 μM/2.5 mM) for 5 min before photobleaching. Distal dendrites of GFP-positive neurons were selected as ROIs (white rectangle) for analysis. FRAP curves for APPL1-GFP fluorescence in total 150 sec are shown. The average fluorescence before photobleaching was counted as 1.0. Gray lines indicate the mean fluorescence intensity ±SEM. Scale bar, 10 μm. (**C**) Dendra2-tagged APPL1 was transfected into hippocampal neurons at DIV 12 for 24 h before photoswitching experiment. Neurons were incubated at 37°C in Tyrode’s solution containing Torlox (10 nM) and subsequently treated with BMI (50 μM). Photoswitch of Dendra2 was performed by UV (405 nm) at the distal dendrites (ROI; red circle) of Dendra2-positive (488 nm) neurons. Images were taken in the red (561 nm) fluorescent channel, and signals were acquired every 1 min for 15 min. Red fluorescence in white-dotted circle indicates nuclear accumulation of APPL1. Scale bar, 100 μm. (**D**) Statistical analysis of somatic APPL1-Dendra2. (**E**) Hippocampal neurons were infected with lentivirus expressing EGFP at DIV 6, and 7 days later, MNI-caged glutamate (200 μM, right panel) or vehicle (as control, left panel) was applied and then uncaged at ROIs (white rectangle) of GFP-positive neurons under UV pulse. After incubation for 30 min, neurons were fixed and stained with antibodies against APPL1 (red) and with DAPI nuclear dye (blue). Scale bar, 10 μm. (**F**) Statistical analysis of nuclear/cytoplasmic ratio of APPL1 with or without glutamate stimulation (* indicates the neuron with caged glutamate; # indicates the neighbor neuron without caged glutamate). (**G** and **H**) Cultured hippocampal neurons were untreated (basal) or treated with Ciliobrevin D (Cilio D, 0.5 mM) or Nocodazole (Noco, 10 μg/ml), respectively, for 1 h. Alternatively, hippocampal neurons were pretreated with Ciliobrevin D or Nocodazole, respectively, for 30 min followed by treatment with BMI for another 1 h. After treatment, the cultures were immunostained with antibodies against MAP2 (green) and APPL1 (color lookup table, pixel intensities from 0 to 255) and with DAPI nuclear dye (blue). Scale bar, 10 μm. Data are presented as mean ± SEM. ***P <* *0.005.

Next, we transfected GFP-tagged APPL1 (APPL1-GFP) into the cultured hippocampal neurons at DIV 12 (12 days *in vitro*) and assayed the movement velocity of APPL1-GFP in the distal dendrites at DIV 14 using fluorescent recovery after photobleaching (FRAP) (Figure 2A and B). Our results revealed that the recovery rate of APPL1-GFP in the distal dendrites was much faster when the neuron was stimulated with BMI/4-AP compared to the unstimulated neurons. These data indicate that the movement velocity of APPL1 in the dendrites is increased following enhanced synaptic activity.

We also monitored the transport of APPL1 using a photoconversible fluorescent protein Dendra2-labelled APPL1 (APPL1-Dendra2) (Figure 2C and D). A brief UV illumination of distal dendrites converted the Dendra2 signal from green to red. Using time-lapse imaging, we followed the transport of photoconverted signals over a period of 30 min postconversion. Our results revealed that the photoconverted (red) dendritic APPL1 underwent stimulus-induced trafficking toward the nucleus.

Furthermore, to test whether endogenous APPL1 undergoes nuclear accumulation following stimulation of distal dendrites, we infected the neurons with a lentivirus expressing EGFP at DIV 6 to visualize the entire dendritic arbor of individual neurons. At DIV 13, neurons were applied with MNI-caged glutamate or vehicle as control, which was then uncaged at the distal dendrites of GFP-expressing neurons by UV illumination, and 30 min later, fixed and immunostained with anti-APPL1 antibodies. We observed that a brief UV pulse at the distal site of the dendrites significantly increased the immunoreactivity of APPL1 in the nucleus as compared to the control (Figure 2E and F), indicating that activation of a subset of synapses in the distal dendrites promotes nuclear APPL1 accumulation.

Finally, pretreatment with Ciliobrevin D to inhibit dynein ATPase or with Nocodazole to induce microtubule depolymerization completely blocked nuclear accumulation of APPL1 induced by BMI/4-AP (Figure 2G and H), indicating that retrograde trafficking of APPL1 is dependent on both dynein and microtubule.

### APPL1 interacts with Importin α1 via its nuclear localization signal

**Figure 3:**
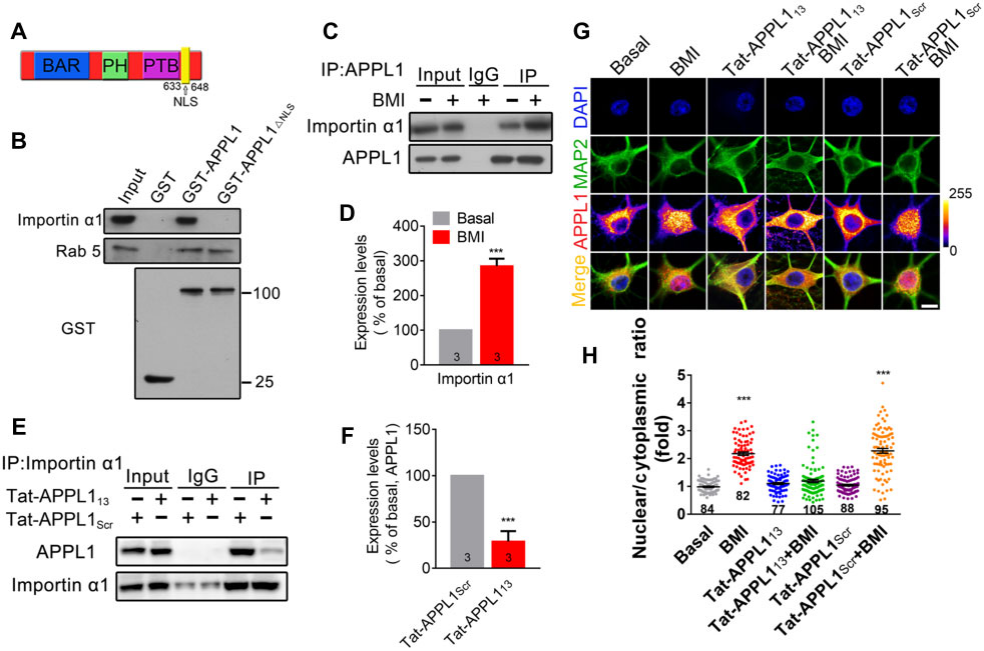
APPL1 contains an NLS. (**A**) Schematic representation of the NLS sequence in APPL1. (**B**) Mouse brain extracts were incubated with recombinant GST, GST-APPL1, or GST-APPL1_ΔNLS_ for 3 h at 4°C. Binding proteins were detected using antibodies against Importin α1 and Rab5, respectively. Recombinant GST, GST-APPL1, or GST-APPL1_ΔNLS_ was detected using antibody against GST. (**C**) Cytosolic fraction from cortical neurons treated with or without BMI was subjected to antibodies against APPL1 for IP and then detected with antibodies against Importin α1 and APPL1, respectively. (**D**) The relative levels of Importin α1 were quantified. (**E** and **F**) Hippocampal neurons treated with Tat-APPL1_13_ or Tat-APPL1_scr_ were subjected to antibodies against Importin α1 for IP and then detected with antibodies against APPL1 and Importin α1, respectively. (**G** and **H**) Hippocampal neurons at DIV 14‒17 were pretreated with Tat-APPL1_13_ (20 μM) or Tat-APPL1_Scr_ (20 μM) for 30 min, followed by incubation with BMI for another 1 h and then immunostaining with antibodies against MAP2 (green) and APPL1 (color lookup table) and with DAPI nuclear dye (blue). Scale bar, 5 μm. The nuclear/cytoplasmic ratio of APPL1 was quantified. ***P <* *0.005 compared to no stimulation. Data are presented as mean ± SEM.

Bigger proteins can be actively transported in the nucleus by dedicated Importins, which recognizes the nuclear localization signal (NLS) located in the protein (Gama-Carvalho and Carmo-Fonseca, 2001; Hanz et al., 2003; Harel and Forbes, 2004). Through bioinformatics analysis, we identified a predicted NLS at the C terminus of APPL1 (APPL1_633__–__648_), implying the binding of APPL1 with Importins (Figure 3A). Consistently, full-length APPL1 (GST-APPL1), but not APPL1 with this sequence deleted (GST-APPL1_ΔNLS_), interacted with Importin α1, while both GST-APPL1 and GST-APPL1_ΔNLS_ interacted with Rab5 (Figure 3B). Furthermore, BMI/4-AP treatment significantly enhanced the interaction between APPL1 and Importin α1, indicating that neuronal activity promotes the recruitment of Importin α1 to APPL1 (Figure 3C and D). Next, we examined whether the interaction between APPL1 and Importin α1 is necessary for nuclear APPL1 translocation. We designed a cell membrane penetrating peptide according to the NLS of APPL1 which was fused with cell membrane transduction domain of trans-activating transcriptional activator from human immunodeficiency virus 1 (Tat-APPL1_13_). Co-immunoprecipitation (co-IP) assay confirmed that pretreatment with Tat-APPL1_13_ blocked the interaction between APPL1 and Importin α1 (Figure 3E and F). Additionally, pretreatment with Tat-APPL1_13_ completely blocked nuclear APPL1 accumulation induced by synaptic activity, whereas pretreatment with scramble peptide (Tat-APPL1_Scr_) had no such effect (Figure 3G and H).

CREB-regulated transcription coactivator 1 (CRTC1) is another protein undergoing nuclear translocation in hippocampal neurons and an NLS is located at its N terminus (Ch’ng et al., 2012; Nonaka et al., 2014). Incubation with Tat-APPL1_NLS_ had no effect on the nuclear accumulation of CRTC1 induced by BMI/4-AP stimulation (Supplementary Figure S2A and B), suggesting that Tat-APPL1_NLS_ specifically blocked the interaction between Importin α1 and APPL1.

### Nuclear APPL1 influences the association of HDAC2 with chromatin

To examine the possible function of nuclear APPL1, we first transfected an NLS-tagged APPL1 into the cultured hippocampal neurons and observed that overexpression of APPL1 in the nucleus had no effect on the abundance of nuclear pERK (Supplementary Figure S3A and B) or pCREB (Supplementary Figure S3C and D), indicating that nuclear APPL1 alone does not induce phosphorylation of nuclear ERK or CREB.

**Figure 4:**
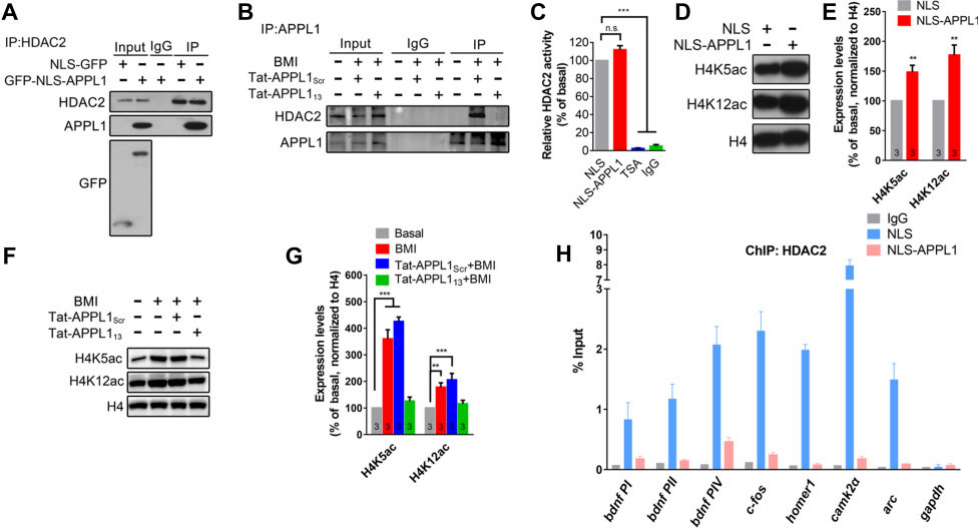
Nuclear APPL1 interrupts the binding of HDAC2 with chromatin. (**A**) PC12 cells were transfected with NLS or NLS-APPL1. After 48 h, cells were harvested and subjected to anti-HDAC2 for IP and detected with anti-APPL1 and anti-HDAC2, respectively. (**B**) Hippocampal neurons were untreated (basal) or pretreated with Tat-APPL1_13_ (20 μM) or Tat-APPL1_Scr_ (20 μM) for 30 min, followed by incubation with BMI (50 μM) for another 30 min at DIV 14‒16, and then collected and subjected to anti-HDAC2 for IP and detected with anti-APPL1 and anti-HDAC2, respectively. (**C**) PC12 cells were transfected with NLS or NLS-APPL. After 48 h, cells were harvested or incubated with TSA (1 μM) for 30 min at DIV 14‒16, immunoprecipitated with anti-HDAC, and then subjected to HDAC2 activity assay. IgG was set as negative control. (**D** and **E**) PC12 cells were transfected with NLS or NLS-APPL1. After 48 h, cells were harvested and detected by western blotting with antibodies against H4K5, H4K12, and histone H4. The relative levels of H4K5 and H4K12 were quantified. **P <* *0.01 compare to NLS group. (**F** and **G**) Hippocampal neurons were untreated (basal) or pretreated with Tat-APPL1_13_ (20 μM) or Tat-APPL1_Scr_ (20 μM) for 30 min, followed by incubation with BMI for another 30 min at DIV 14‒16, and then collected and detected by western blotting with antibodies against H4K5, H4K12, and histone H4. The relative levels of H4K5 and H4K12 were quantified. (**H**) PC12 cells were transfected with NLS or NLS-APPL1. After 48 h, cells were harvested and subjected to anti-HDAC2 for ChIP. The specificity of DNA binding for HDAC2 was quantified according to the RT–qPCR signals. **P <* *0.01, ***P <* *0.005 compare to basal group. n.s., not significant. Data are presented as mean ± SEM.

Next, we focused on histone deacetylases (HDAC1/2) which have been identified as binding partners of nuclear APPL1 via proteomic analysis (Miaczynska et al., 2004). We transfected GFP-NLS-APPL1 into the PC12 cells and the interaction of nuclear APPL1 with HDAC2 was observed via co-IP (Figure 4A). Moreover, the interaction between endogenous APPL1 with HDAC2 was detected in the cultured neurons treated with BMI/4-AP for 30 min, which was completely blocked by pretreatment with Tat-APPL1_13_, but not with scramble peptide (Figure 4B). However, no interaction was observed between GST-APPL1 and HDAC2 (Supplementary Figure S4), indicating that APPL1 has no direct interaction with HDAC2.

To test whether nuclear APPL1 affects HDAC2 activity, PC12 cells were transfected with NLS-APPL1 or NLS only, cell lysates were immunoprecipitated with an HDAC2 antibody, and HDAC2 activity was assessed using a fluorimetric assay. As shown in Figure 4C, no difference in HDAC2 activity was detected between cells transfected with NLS-APPL1 or NLS, indicating that APPL1 has no effect on HDAC2 activity.

To test the effect of nuclear APPL1 on histone acetylation, PC12 cells were transfected with NLS-APPL1 and acetylation of histone H4 was detected. As shown in Figure 4D and E, the acetylation levels of H4 at the H4K5 and H4K12 sites were significantly increased after overexpression of APPL1 in the nucleus. Furthermore, treatment with BMI/4-AP induced rapid acetylation of histone H4 in the cultured hippocampal neurons, and this effect was completely blocked by pretreatment with Tat-APPL1_13_, but not with scramble peptide (Figure 4F and G). In the following, we performed chromatin immunoprecipitation (ChIP) assays using PC12 cells to test whether nuclear APPL1 affects the binding ability of HDAC2 to chromatin. We surveyed a total of five genes, which are regulated by neuronal activity and contain a CREB/HDAC2 binding site in their promoters. As shown in Figure 4H, the association of HDAC2 with the promoters for these genes was significantly attenuated in PC12 cells transfected with NLS-APPL1, compared to the control group expressing empty vector. Taken together, these results indicate that nuclear APPL1 participates in chromatin remodeling via interrupting the interaction between HDAC2 and chromatin.

### Nuclear translocation of APPL1 regulates gene transcription

**Figure 5:**
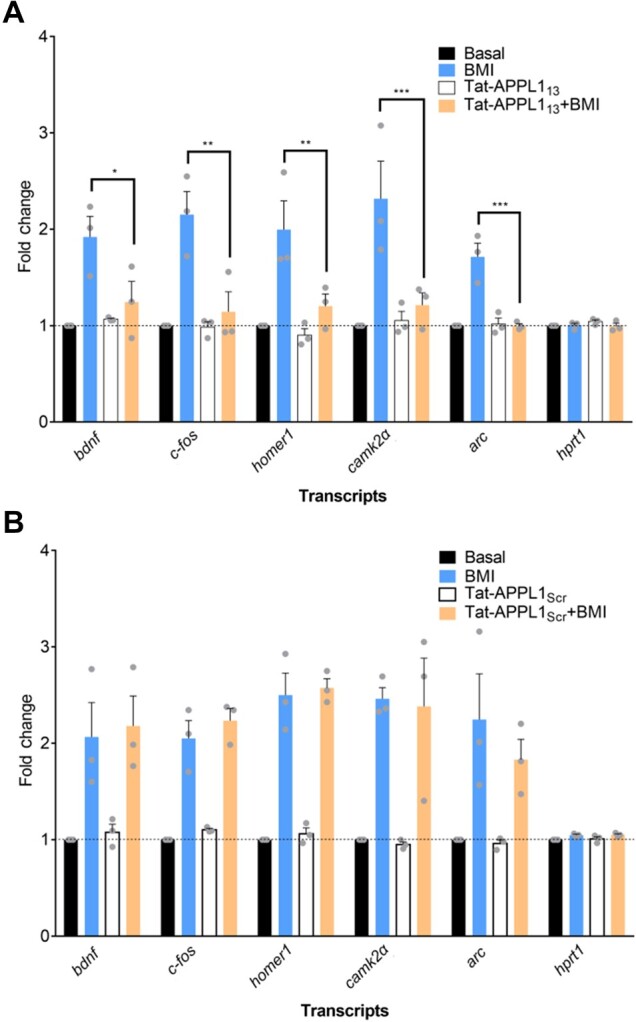
Pretreatment with Tat-APPL1_13_ influences synaptic activity-induced gene transcription. Hippocampal neurons at DIV 14‒17 (**A**) or hippocampal neurons (**B**) were untreated, treated with BMI or Tat-APPL1_13_ (20 μM) for 1 h, or pretreated with Tat-APPL1_13_ for 30 min followed by BMI for another 1 h. mRNA levels of CREB-related genes were measured by qRT–PCR.

Next, to examine whether nuclear translocation of APPL1 is necessary for the regulation of gene expression, we analysed several genes, including *bdnf, c-fos, homer1, camk2a*, and *arc*, which were reported to be transcribed at early phase of synaptic plasticity (Zhang et al., 2009). Here, RNA isolated from the hippocampal neurons was used for quantitative reverse transcriptase (RT–qPCR) analysis. Incubation with BMI/4-AP significantly increased the transcription levels of most of the above-mentioned genes, and pretreatment with Tat-APPL1_13_ (Figure 5A), but not with Tat-APPL1_Scr_ (Figure 5B), completely blocked these effects.

### Nuclear translocation of APPL1 is critical for the maintenance of late-phase long-term potentiation

**Figure 6:**
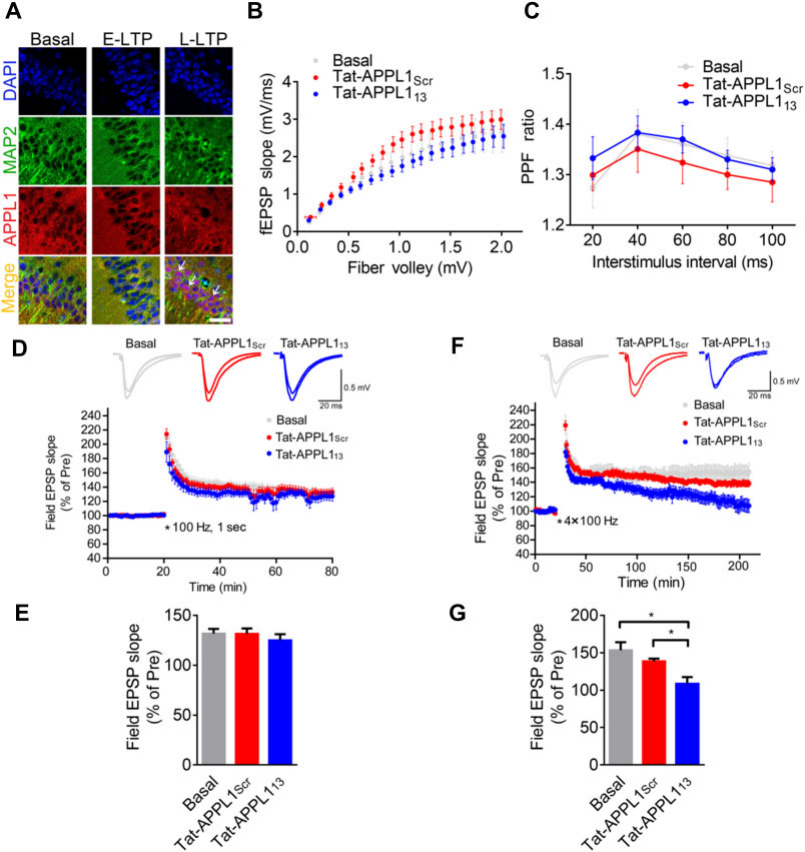
Retrograde translocation of APPL1-positive TrkB endosomes is required for L-LTP. (**A**) E-LTP was induced by a single train of 100 Hz in acute hippocampal slices from wild-type mice and L-LTP was induced by four trains of 100 Hz every 3 min and subsequently incubated for 30 min in ACSF at 32°C. Slices were fixed and stained with antibodies against MAP2 (green) and APPL1 (red) and with DAPI nuclear dye (blue). Arrowheads indicate nuclear accumulation of APPL1. Scale bar, 50 μm. (**B**) The input‒output curve at hippocampal Schaffer collateral-CA1 synapses was unchanged when bath-applied with Tat-APPL1_13_ (*n *=* *6 slices, 4 mice) compared to untreated (basal, *n *=* *5 slices, 3 mice) or treated with Tat-APPL1_Scr_ (*n *=* *6 slices, 4 mice). (**C**) The paired-pulse ratio was unchanged when bath-applied with Tat-APPL1_13_ (*n *=* *5 slices, 3 mice) compared to untreated (basal, *n *=* *5 slices, 3 mice) or treated with Tat-APPL1_Scr_ (*n *=* *5 slices, 3 mice). (**D** and **E**) E-LTP was induced by one train of 100-Hz simulation (100 Hz, 1 sec). Perfusion with Tat-APPL1_13_ has no effect on the maintenance (last 10 min, *n *=* *7 slices, 4 mice) of E-LTP compared to the basal group (*n *=* *9 slices, 5 mice) or Tat-APPL1_Scr_ group (*n *=* *9 slice, 5 mice). Scale bars, 20 ms, 0.5 mV. (**F** and **G**) L-LTP was induced by four trains of tetanic stimulation (3 min apart). Perfusion with Tat-APPL1_13_ significantly decreased the maintenance of L-LTP (*n *=* *8 slices, 8 mice) compared to basal group (*n *=* *7 slices, 7 mice), while perfusion with Tat-APPL1_Scr_ (*n *=* *6 slices, 6 mice) showed no effect (last 10 min). Sample traces 10 min before and 1 or 3 h after the tetanus. Scale bars, 20 ms, 0.5 mV. Data are presented as mean ± SEM.

Among the genes analysed in Figure 5, *Bdnf* and *camk2a* are genes tightly related to synaptic plasticity. Next, we examined whether nuclear translocation of APPL1 plays a role in synaptic plasticity. Immunostaining assay revealed that nuclear accumulation of APPL1 occurred in parts of hippocampal pyramidal neurons of the brain slices induced by four trains of tetanic stimulation, but not in that of the brain slices induced by a single train of stimulation (Figure 6A). We then measured synaptic transmission at hippocampal Schaffer collateral-CA1 pyramidal (SC-CA1) synapses. Basal excitatory transmission, such as input–output (Figure 6B) and paired-pulse ratio (Figure 6C) was unchanged when bath-application with Tat-APPL1_13_ (20 μM) compared to Tat-APPL1_Scr_ (20 μM). Early-phase long-term potentiation (E-LTP) was successfully induced by a single train of 1 sec, 100-Hz tetanic stimulation and bath-applied Tat-APPL1_13_ had no effect on the induction and maintenance of E-LTP compared to Tat-APPL1_Scr_ (Figure 6D and E), indicating that nuclear translocation of APPL1 is not involved in the E-LTP. Next, late-phase LTP (L-LTP) was induced by four trains of 100-Hz tetanic stimulation and the enhancement of field EPSP could maintain at least 3 h. Infusion with Tat-APPL1_Scr_ peptide (138.4% ± 3.7%, *n *=* *6) had no effect on the induction or maintenance of L-LTP compared to the untreated group (153.2% ± 10.8%, *n *=* *7). In contrast, infusion with Tat-APPL1_13_ significantly impaired the maintenance of L-LTP (108.4% ± 9.0%, *n *=* *8) compared to the untreated or Tat-APPL1_Scr_-treated group, while it had no effect on the induction of L-LTP (Figure 6F and G; Supplementary Figure S5). Taken together, these data indicate that nuclear translocation of APPL1 is required for the maintenance of L-LTP but not that of E-LTP.

## Discussion

In this study, we demonstrated that adaptor protein APPL1 shuttles into the nucleus under stimulation and promotes histone acetylation, and this process is required for the activity-induced gene transcription and the maintenance of LTP. Therefore, APPL1 acts as a linker coupling neuronal activity with gene transcription, and this pathway contributes to the modification of synaptic plasticity.

Previous researches have observed the nuclear translocation of APPL1 in non-neuronal cells (Miaczynska et al., 2004). Here, we confirmed the activity-induced nucleocytoplasmic shuttling of APPL1 in both cultured hippocampal neurons (Figure 1) and in acute brain slice (Figure 6). To analyse the causal role of nuclear APPL1 in gene transcription, we utilized a transmembrane peptide to block the interaction between APPL1 and Importin α1, instead of changing the expression level of APPL1 (Banach-Orlowska et al., 2009, 2015; Rashid et al., 2009). It should be kept in mind that APPL1 is a multifunctional protein and synaptic APPL1 has been identified to couple NMDA receptors with AKT signaling (Wang et al., 2012) and gate LTP (Fernandez-Monreal et al., 2016). It is better to specifically interrupt the nuclear translocation of APPL1, while leaving its other functions unaffected.

In our study, we observed that enhanced neuronal activity triggers retrograde translocation of APPL1 along the dendrites (Figure 2), indicating that nuclear-accumulated APPL1 is mainly from the dendrites. However, we cannot completely exclude the possibility that retrograde translocation of APPL1 along the axon may also contribute to nuclear APPL1 accumulation. Especially, Zerial’s group has shown data indicating that APPL1 undergoes both retrograde and anterograde trafficking along the axon under basal conditions (Goto-Silva et al., 2019). It is interesting to differentiate the functional roles of dendritic transport of APPL1 from that of axonal transport in future.

Similar to APPL1, several endocytic proteins involved in vesicle trafficking and sorting, such as clathrin (Borlido et al., 2009), β-arrestin1 (Beaulieu and Caron, 2005), Huntingtin interacting protein 1 (Mills et al., 2005), and intersectin 1-short (Alvisi et al., 2018), are capable of shuttling between the nucleus and the cytoplasm and being involved in nuclear signaling in response to extracellular stimuli (Pilecka et al., 2007; Borlido et al., 2009). It is still unclear to what extent these diverse functions are interconnected to coordinate various cellular processes, or whether they are largely independent. At least, these findings suggest that nuclear translocation of endocytic proteins may represent a powerful channel for communicating information from the extracellular environment to the nucleus. Moreover, endocytosis mediated by APPL1/Rab5 constitutes a novel APP-dependent pathogenic pathway in Alzheimer’s disease (AD) (Kim et al., 2016) and APPL1 has also been found to accumulate as granules around neurons in postmortern human brain of AD (Ogawa et al., 2013). Thereafter, endocytic protein-mediated pathway should be a potentiated target to unveil the pathophysiology of these neurodegenerative diseases in future studies.

## Materials and methods

### Animals

Mice were housed under a 12-h light and dark cycle with food and water provided ad libitum. All experiments were performed in accordance with the guidelines of Zhejiang University Animal Experimentation Committee. Male C57BL/6 mice (6‒8 months of age) were used in related experiment.

### Plasmids

GST-APPL1 and GST-APPL1_ΔNLS_ were constructed by cloning the corresponding cDNA of mouse APPL1 into pGEX-4T-1 between *Xho*I and *Eco*RI. FLAG-NLS-APPL1 was constructed by cloning the cDNA of human APPL1 into pCMV-Tag4A vector between *Eco*RV and *Xho*I. NLS-GFP, GFP-NLS-APPL1, or Importin α1 was constructed by cloning the cDNA into pEGFP or pERFP vector between *Xho*I and *Eco*RI. APPL1-Dendra2 was constructed by cloning the human APPL1 into pCMV-Dendra2 between *Eco*RI and *Xho*I.

### Reagents, peptides, and antibodies

4-Aminopyridine, PTX, poly-L-lysine, D(−)-2-amino-5-phosphonopentanoic acid, Trolox, DAPI nuclear dye, Nocodazole, and Ciliobrevin D were purchased from Sigma. Bicuculline methiodide, MNI-caged-L-glutamate, Anisomycin were purchased from Tocris Bioscience. Leptomycin B (10 nM) was purchased from Beyotime. Peptides used are Tat-APPL1_13_ (YGRKKRRQRRRRRASEKQKEIERVKEK) and Tat-APPL1_Scr_ (YGRKKRRQRRRRRASEKQKEIEAAAAA). The following antibodies were used: anti-APPL1 (sc-67402) and anti-Rab5 (sc-46692) from Santa Cruz Biotechnology, anti-pERK (4370S) and anti-GAPDH (2118) from Cell Signaling Techonology, anti-HDAC2 (ab32117), anti-Importin α1 (ab84440), anti-Histone H4K5 (ab51997), anti-Histone H4K12 (ab177793), anti-APPL2 (ab95196), and anti-Histone H4 (ab10158) from Abcam, anti-MAP2 (M9942, M3696), anti-FLAG (F1804), and anti-APPL1 (1409089) from Sigma–Aldrich, and anti-pCREB (06-519 and 04-218) from Millipore. Glutathione sepharose beads and protein A sepharose beads were purchased from GE Healthcare. Phenylmethylsulfonyl fluoride (PMSF) and phosphatase inhibitor cocktails 2 and 3 were purchased from Sigma. Horseradish peroxidase (HRP)-linked goat anti-mouse immunoglobulin G (IgG), goat anti-rabbit IgG, and donkey anti-goat IgG, secondary antibodies conjugated to Dylight (488 or 555), and chemiluminescence kit were purchased from Pierce.

### Cell cultures and transfection

PC12 cell line was grown on Dulbecco’s modified Eagle media (DMEM, Gibco) supplemented with 10% fetal bovine serum (Gibco), and 1% penicillin/streptomycin (Gibco) at 37°C under 5% CO_2_. Hippocampal or cortical neurons were cultured as described previously (Luo et al., 2002; Wang et al., 2012). Hippocampal neurons were transfected with the plasmids using Lipofectamine 3000 reagent (Life Technologies) at DIV 10‒13 or Calcium Phosphate Transfection Kit (Clontech) at DIV 6‒9 according to the manufacturer’s instructions.

### Pharmacological treatment

For most of the experiments, neurons were incubated with various pharmacological regents in cultured medium in a 37°C, 5% CO_2_ incubator for the appropriate amount of time before cells were either fixed for immunostaining or lysates were collected for western blotting. For extrasynaptic NMDAR activation, cultures were first stimulated by BMI/4-AP in the presence of the non-competitive NMDAR antagonist MK801 (10 μM) for 5 min to inactivate synaptic NMDARs. After washout, extrasynaptic NMDAR were then selectively activated by NMDA (10 μM) for 1 h. KCl depolarization was induced in solutions containing 140 mM NaCl, 1.3 mM CaCl_2_, 50 mM KCl, 35 mM HEPES, and 33 mM glucose (pH 7.4) for 30 min. When peptides or antagonists were used to pretreat the cultured neurons, these reagents were applied 30 min before stimulation unless otherwise indicated. When peptides or antagonists were used to pretreat the cultured neurons, these reagents were applied 30 min before stimulation unless otherwise indicated.

### Immunostaining

Hippocampal neurons were fixed at room temperature with 4% paraformaldehyde (PFA) in phosphate-buffered saline (PBS) for 15 min, washed three times in PBS, permeabilized with 0.2% Triton X-100 (Amresco) in PBS, and then blocked for 30 min in blocking solution containing 2.5% BSA fraction V (Amresco) in PBS. Next, neurons were incubated in primary antibodies for 1 h at room temperature or overnight at 4°C, washed three times with PBS, and then incubated with secondary antibody in blocking solution for another 1 h at room temperature. After being washed, neurons were mounted with ProLong Gold or ProLong Gold with DAPI (Molecular Probes).

Acute brain slices (50 μm) or frozen sections (25 μm) were fixed with 4% PFA overnight at 4°C, permeabilized with 0.4% Trition X-100 in PBS for 30 min at room temperature, and then blocked in 2.5% BSA for 2 h at room temperature. Slices were incubated with primary antibodies for 48 h at 4°C. After being washed with PBS, slices were then incubated with an appropriate secondary antibody and nucleus dye DAPI for 4 h at room temperature. Slices were mounted with ProLong Gold.

### Recombinant protein purification and pull-down assay

GST-APPL1 or GST-APPL1_ΔNLS_ was transformed in *Escherichia coli* BL21 and induced with 0.1 mM isopropyl β-D-thiogalactopyranosideI (IPTG) for 6 h at 30°C. The recombinant APPL1 and APPL1_ΔNLS_ were purified using glutathione-sepharose beads. To pull down proteins from brain lysates, fresh mouse brain was homogenized in lysis buffer (50 mM HEPES, 100 mM NaCl, 1 mM EDTA, 1% Triton X-100, pH 7.6) with protease and phosphatase inhibitors. After homogenization, the mixture was incubated for 30 min at 4°C, and then centrifuged at 16000× *g* for 15 min at 4°C. Subsequently, 500 μl supernatant was incubated with 10 μg purified recombinant proteins for 3 h at 4°C. The complex was washed four times with lysis buffer and subjected to western blotting analysis. For binding assay, 10 μg purified GST-tagged protein was incubated with approximately the same amount of His-tagged recombinant protein in lysis buffer. Proteins were pulled down with GST beads, washed four times with lysis buffer, and then detected with His antibody.

### Quantitative real-time PCR

Total RNA was extracted from cortical neurons using Trizol (TaKaRa) and reversely transcribed into cDNA with PrimeScript RT Reagent Kit (TaKaRa) according to the manufacturer’s instructions. qRT–PCR was carried out using CFX96 Real-Time PCR Detection System (Bio-Rad). Gene expression levels were calculated according to the 2^−^^ΔΔCt^ method. The relative amounts of mRNA were normalized to *β-actin* as an internal control, and *hprt1*, an activity-independent gene, was set as a negative control. Primer sequences were as follows (Sequence 5′–3′): *camkIIα*-F, ACCTGCACCCGATTCACAG; *camkIIα*-R, TGGCAGCATACTCCTGACCA; *hprt1*-F, TGTTGTTGGATATGCCCTTG; *hprt1*-R, GGCCACAGGACTAGAACACC; *arc*-F, AAGTGCCGAGCTGAGATGC; *arc*-R, CGACCTGTGCAACCCTTTC; *bdnf*-F, ACGACATCACTGGCTGACACT, *bdnf*-R, GAAAGAGTAGAGGAGGCTCCAA; *c-fos*-F, CACACAGGACTTTTGCGC, *c-fos*-R, GACACGGTCTTCACCATTCC; *homer1*-F, CAACAGCTTGCTGCGTACC; *homer1*-R, CTAACACACTCCAGCTCAGTGAC; *β-actin*-F, CCAACTGGGACGATATGGAGAAGA; *β-actin*-R, CGCA CGATTTCCCTCTCAGC.

### Time-lapse live-cell imaging

For the live-cell imaging, hippocampal neurons were transfected with APPL1-GFP or APPL1-Dendra2 at DIV 12 and imaged 2 days later. At 1 h before imaging, cultured medium was replaced with Tyrode’s solution (25 mM HEPES buffer, PH 7.4, 119 mM NaCl, 5 mM KCl, 2 mM CaCl_2_, 2 mM MgCl_2_, and 10 mM glucose) with Trolox (10 nM) using an Olympus FV1000 confocal microscope equipped with a Disk Scanning Unit, an EM-CCD, and 40×/1.35 NA oil or 60×/1.35 NA oil under 512 × 512 pixels resolution at a scanning zoom of 5.0 in the x-direction. Temperature was maintained at 37°C in 5% CO_2_ environmental chamber and Z-Drift Compensator (ZDC) system was used to correct loss of focus. Moreover, 2 μs/pixel and <15% laser power were used to minimize photobleaching during acquisition. Images were taken every 5 sec for FRAP experiments and were captured every 1 min for photoswitch of APPL1-Dendra2 at the same laser power conditions.

APPL1-GFP within rectangular region of interest (ROI, 10 μm × 20 μm) along the dendritic branch or distal axons was photobleached by scanning with the 405 nm line of laser at 100% intensity for 800 μs. Average fluorescence in ROIs was measured, background was subtracted, and images were corrected for overall photobleaching in each time frame. We measured the recovery mobile fraction of bleached area by fitting equation: *F*_m_=*F*_t_ − *F*_0_/*F*_i_ − *F*_0_, where *F*_m_ was the mobile fraction, *F*_t_ was the end value of the recovery intensity, *F*_i_ was the initial value of the area before bleaching, and *F*_0_ was the background fluorescence intensity. The analysis of fluorescence was performed using ImageJ software (National Institutes of Health).

Photoswitch of APPL1-Dendra2 within circle ROI (*r* = 25 μm) along the dendritic branch or distal axons was performed using 405 nm line of laser with 100% intensity for 200 μs. Images were acquired using 488 nm line of laser before photoswitch and 561 nm line of laser after switch. Subsequently, image analysis was carried out with the ImageJ and FV10-ASW2.0 software (Olympus).

### Slice preparation and electrophysiology

C57BJ/6 mice at 8‒12 weeks of age were anesthetized with diethyl ether, and the brains were rapidly removed and placed in ice-cold, high sucrose cutting solution containing 194 mM sucrose, 30 mM NaCl, 26 mM NaHCO_3_, 10 mM glucose, 4.5 mM KCl, 1.2 mM NaH_2_PO_4_, 7 mM MgSO_4_, 0.2 mM CaCl_2_, and 2 mM MgCl_2_. Slices were cut on a Leica vibratome in the high sucrose cutting solution and immediately transferred to an incubation chamber with artificial cerebrospinal fluid (ACSF) containing 119 mM NaCl, 26.2 mM NaHCO_3_, 11 mM glucose, 2.5 mM KCl, 1 mM NaH_2_PO_4_, 1.3 mM MgCl_2_, 11 mM glucose, and 2.5 mM CaCl_2_. The slices were allowed to recover at 34°C for 30 min before being allowed to equilibrate at room temperature for another 1 h. During recordings, the slices were placed in a recording chamber constantly perfused with heated ACSF (28°C‒32°C) and gassed continuously with 95% O_2_ and 5% CO_2_. Extracellular field EPSPs (fEPSPs) were recorded in stratum radiatum of CA1 using a glass pipette filled with ACSF (1.5‒3 mΩ). The SC pathway was stimulated every 20 sec using a concentric bipolar stimulation electrode. For LTP, the stimulation intensity was adjusted to give fEPSP slopes of 30%‒50% of maximum, and three successive responses were averaged and expressed relative to the normalized baseline. After a stable baseline was recorded, one train of 100-Hz (100 Hz, 1 sec) or four trains of 100-Hz (3 min apart) stimulation were applied to induce E-LTP or L-LTP. Peptides (20 μM) were bath-applied for >30 min before the LTP protocol application. For immunohistochemistry, brain slices induced by high-frequency stimulation were kept in ACSF for another 30 min and then immunostained accordingly.

### Statistical analysis

No statistical methods were used to pre-determine sample sizes, but our sample sizes were similar to those reported in previous studies. Samples were assigned randomly to the experimental and control groups. Experimental treatments were also randomized. All data are shown as the mean ± the standard error of the mean (SEM). Differences between two groups were tested by unpaired, two-tailed Student’s *t*-test or Mann–Whitney rank sum test, based on a normality test (Shapiro–Wilk). For comparison of more than two groups, one-way analysis of variance (ANOVA) and Bonferroni’s test for multiple comparison *post hoc* tests were used. We used two-way ANOVA and Tukey test for *post hoc* test if there were two independent variables. Statistical analysis was carried out using Prism 5 software (GraphPad). Significance was indicated as ***P <* *0.005, **P <* *0.01, and *P <* *0.05.

## Supplementary material

Supplementary material is available at *Journal of Molecular Cell Biology* online.

## Supplementary Material

mjaa058_Supplementary_Data
