# Supplementary material for: Adaptor protein APPL1 links neuronal activity to chromatin remodeling in cultured hippocampal neurons
Source: J Mol Cell Biol. 2020 Oct 26;13(5):335–46. doi: 10.1093/jmcb/mjaa058 (PMC8373263; doi:10.1093/jmcb/mjaa058)
Supplement: mjaa058_Supplementary_Data [file mjaa058_supplementary_data.pdf]

Supplementary Materials

Adaptor protein APPL1 links neuronal activity to chromatin remodeling in cultured hippocampal neurons

Yu Wu[1], Xinyou Lv[1], Haiting Wang[1], Kai Qian[1], Jinjun Ding[1], Jiejie Wang[1], Shushan Hua[1], Tiancheng Sun[1], Yiting Zhou[3], Lina Yu[1], and Shuang Qiu[1,2]*

[1]Department of Neurobiology and Department of Anesthesiology of Second Affiliated Hospital, Zhejiang University School of Medicine, Hangzhou 310058, China.

[2]NHC and CAMS Key Laboratory of Medical Neurobiology, MOE Frontier Science Center for Brain Research and Brain-Machine Integration, School of Brain Science and Brain Medicine, Zhejiang University, Hangzhou 310058, China.

[3]Department of Biochemistry and Department of Orthopaedic Surgery of the Second Affiliated Hospital, Zhejiang University School of Medicine, Hangzhou 310058, China.

***To whom correspondence should be addressed: Dr. Shuang Qiu**, E-mail: qiushly@zju.edu.cn .

The authors have no conflict of interest

Supplementary Material and Methods

Co-immunoprecipitation and Western Blot

Co-immunoprecipitation (Co-IP) was carried out as reported previously (Qiu et al., 2014). Briefly, neurons were lysed in a radio-immunoprecipitation (RIPA) assay buffer [50 mM Tris-HCl (pH 7.4), 150 mM NaCl, 1% NP-40, 0.25% sodium deoxycholate, 2 mM EDTA, 0.1mM PMSF], supplemented with phosphatase inhibitor cocktails 2 and 3. After centrifuged (12,000g, at 4°C) for 30 min, the pellet was discarded, and the supernatant was preincubated with IP antibody or IgG overnight at 4°C. Protein A-Sepharose was then added and incubated for another 2 hr at 4°C. The mixtures were washed three times with RIPA assay buffer, eluted by boiling in 2x Laemmli buffer, and subjected to western blot analysis with the antibodies accordingly.

Western blot was conducted as described previously (Qiu et al., 2013; Wang et al., 2018). Protein samples were performed on SDS-polyacrylamide gels, and separated proteins was transferred to Nitrocellulose Membrane (Whatman, GE Healthcare). The membranes were blocked with 5% BSA in Tris-buffered saline with Tween-20 (TBST) at room temperature for 2 hr and were incubated with primary antibodies overnight at 4°C. After being washed three times with TBST, the membranes were incubated with appropriate HRP-linked secondary antibody (1:10000) for 1 hr at room temperature, followed by detection of the proteins with the chemiluminescence reagent according to the instructions of the manufacturer. The density of the western blot was measured with Quantity One under GS-800 Calibrated Densitometer (Bio-Rad).

Extraction of histone proteins

Histone fractionation was performed as described previously with a slightly modified (Shechter et al., 2007). In brief, nuclear fraction was prepared with Nuclear Extract Kit (Active Motif) according to the manufacturer's instructions. Re-suspend nuclei in 400 µl 0.4N H_2SO_4 and incubate on rotator for 2 hr at 4°C. After incubation, samples were centrifuged at 16,000 g for 10 min at 4°C to remove nuclear debris. Then add 132 µl TCA drop by drop to histone solution and invert the tube several times to mix the solutions (final concentration of TCA is 33%). Samples were incubated on ice for 1 hr and centrifuged at 16,000 g for 10 min at 4°C, histone pellet were air-dry for 20 min at room temperature then re-suspend with appropriate volume of ddH_2O.

Chromatin immunoprecipitation (ChIP)

ChIP assays were performed using the EZ-ChIP assay kit (Millipore) following the manufacturer's protocol. Briefly, PC12 cells were transfected with NLS or NLS-APPL1 for 48 hr. Cells were cross-linked with 1% formaldehyde for 10 min at 37 °C. Crosslinking was stopped with 0.125M glycine. After sonication to yield DNA fragments of 200-500 base pairs, the lysates were cleared by centrifugation, diluted 6-fold with ChIP dilution buffer, and precleared with salmon sperm DNA/protein A-agarose at 4°C for 1 hr. 1% input. For each immunoprecipitation assay, the lysates were incubated with 2 µg of, anti-HDAC2 or control IgG (Thermo Fisher) overnight at 4°C with rotation. The immune-complexes were then collected with protein A-agarose slurry, eluted, and de-crosslinked at 65 °C. After RNase digestion and proteinase

digestion, immunoprecipitated DNA was extracted (QIAGEN). The purified DNA was amplified by real time qPCR. The relative amounts of mRNA were normalized to *β-actin* as an internal control, and *hprt1,* an activity-independent gene, was set as a negative control. Primer sequences were as following (Sequence 5'-3'):

camkIIα-F, GACCTGGATGCTGACGAAGR; *camkIIα*-R, AGGTGATGGTAGCCAT CCTG; *bdnf* PI-F, TGATCATCACTCACGACCACG; *bdnf* PI, CAGCCTCTCTGAGCC AGTTACG; *bdnf* PII-F, TGAGGATAGTGGTGGAGTTG; *bdnf* PII, TAACCTTTTCCTC CTCC; *bdnf* PIV-F, GCGCGGAATTCTGATTCTGGTAAT R; *bdnf* PIV, GAGAGGGCTC CACGCTGCCTTGACG; *arc*-F, CAGCATAAATAGCCGCTGGT; *arc*-R, GAGTGT GGCAGGCTCGTC; *gapdh*-F, CTCCCAGGAAGACCCTGCTT; *gapdh*-R, GGAACAG GGAGGAGCAGAGA; *c-fos*-F, GAAAGCCTGGGGCGTAGAGT, *c-fos*-R, CC TCAGCTGGCGCCTTTAT; *homer1*-F, CTGCCTGAGTGTCGTGGAAG; *homer1*-R, ATGATTTCACTCGCGCTGAC.

HDAC activity assay

HDAC activity was measured using the HDAC2 Immunoprecipitation (IP) & Activity Assay Kit according to the instructions from the manufacturer (BIOVISION). Briefly, PC12 cells were transfected with NLS or NLS-APPL1 or 1 μm Trichostatin A (TSA, MCE). Then immunoprecipitates with anti-HDAC2 in a rotary mixer at 4°C overnight (IgG was set as negative control), Protein A/G were added into samples for 2hr incubation at 4°C. HDAC2 assay buffer was added into tube for 2 hr incubation at room temperature and stopped by adding developer solution. The fluorescence of the

substrate was measured after 30 min at 380 nm excitation/500 nm emission using a spectral scanning multimode reader (ThermoFisher Varioskan Flash).

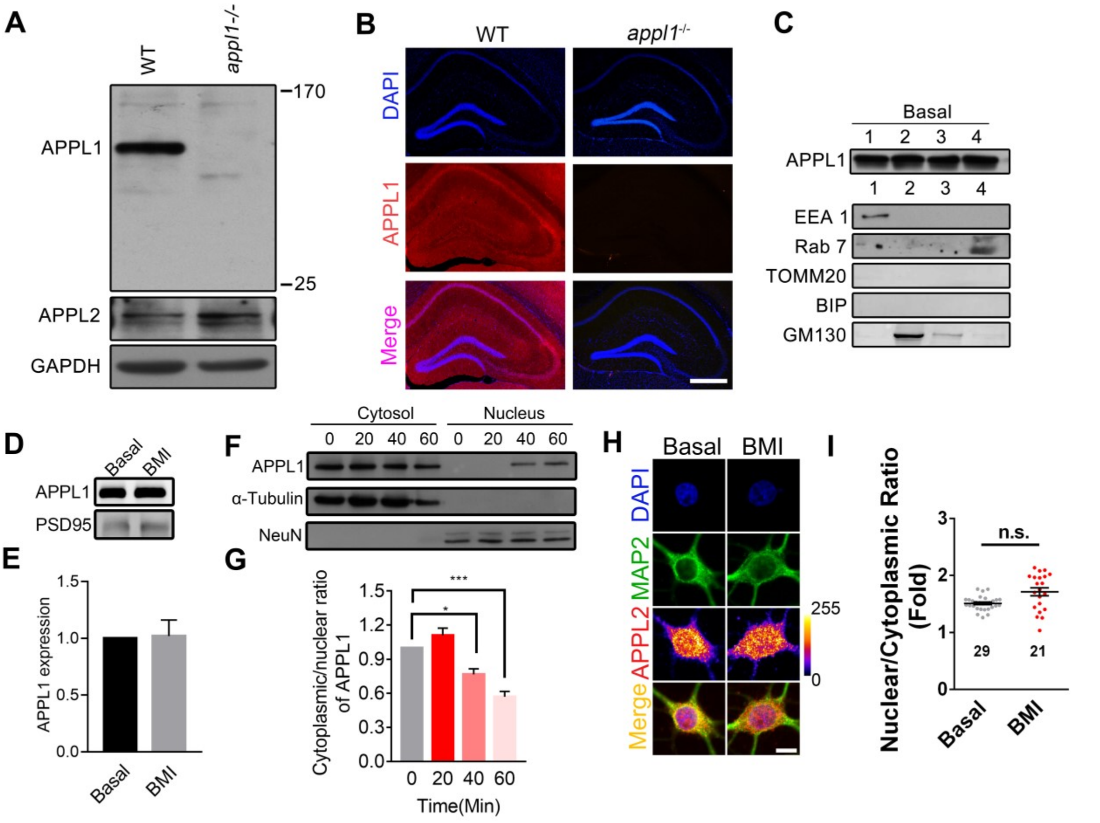

Supplementary Figure 1 Neuronal activity has no effect on nuclear accumulation of APPL2.

(A) Hippocampal tissues from wild type (WT) or conventional APPL1 knockout (*appl1-/-*) mice were homogenized and detected by western blot with antibodies against APPL1, APPL2, and GAPDH. **(B)** Immunohistochemical staining of APPL1 and DAPI in hippocampal slices from wild type (WT) or *appl1-/-* mice. **(C)** Cortical neurons were subjected to subcellular fractionation at DIV 10-12. Cells were extracted after centrifugation with an Optiprep discontinuous gradient and analyzed by immunoblotting with antibodies against EEA1 (Early endosome, Fraction 1), Rab7 (Late endosome, Fraction 4), TOMM20 (Mitochondria), BIP (Endoplasmic reticulum),

GM130 (Golgi, Fraction 2 and 3), and APPL1, respectively. **(D and E)** The abundance of APPL1 in the cultured cortical neurons after BMI/4-AP treatment for 1 hr. **(F and G)** The abundance of APPL1 in the cytosolic and nuclear fractions of the cultured cortical neurons after BMI/4-AP treatment for indicated time. **(H and I)** Hippocampal neurons at DIV 14-17 were treated with BMI (50 μM) for 1 hr and subsequently stained with anti-MAP2, anti-APPL2 and DAPI nuclear dye. Scale bar, 25 μm. Stained APPL2 was represent by color lookup table. The nuclear/cytoplasmic ratio were quantified. All data are plotted Mean ±SEM. n.s., not significant.

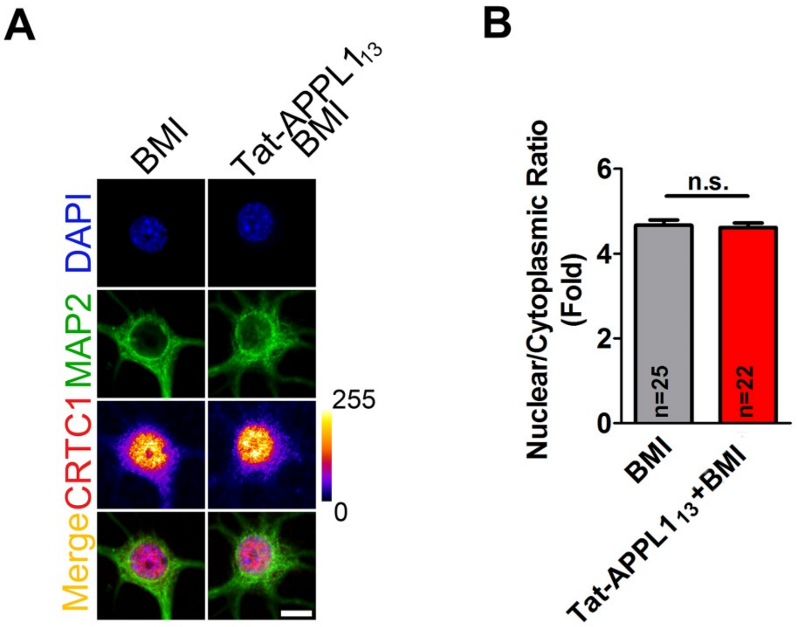

Supplementary Figure 2 Pretreatment with Tat-APPL1$_{NLS}$ has no effect on nuclear translocation of CRTC1.

(A) Hippocampal neurons at DIV 14-17 were pretreated with Tat-APPL1$_{13}$ (20 μM) and subsequently treated with BMI (50 μM) for 1 hr, followed by staining with anti-MAP2, anti-CRTC1 and DAPI nuclear dye. Scale bar, 25 μm. Stained CRTC1 was represent by color lookup table. **(B)** The nuclear/cytoplasmic ratio were quantified. All data are plotted Mean ±SEM. n.s., not significant.

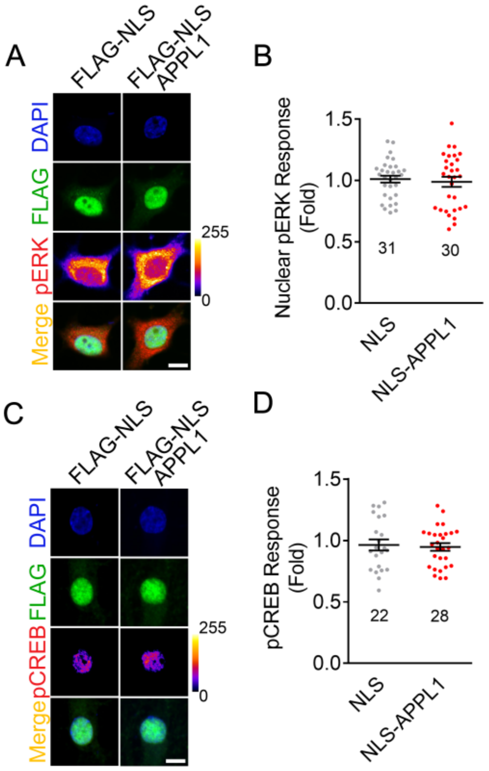

Supplementary Figure 3 Nuclear accumulation of APPL1 has no effect on nuclear ERK/CREB activity.

(A and B) Hippocampal neurons transfected with FLAG-NLS or FLAG-NLS-APPL1 were labeled with antibodies against FLAG (green) and pERK (color lookup table) and with DAPI. The fluorescence intensity of nuclear pERK was then quantified. Scale bar = 10 μm. **(C and D)** Neurons transfected with FLAG-NLS or FLAG-NLS-APPL1 were labeled with antibodies against FLAG (green) and pCREB (color lookup table) and with DAPI (blue). The fluorescence intensity of pCREB was then quantified. Scale bar = 10 μm.

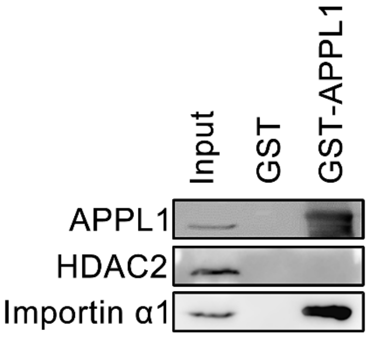

Supplementary Figure 4 APPL1 has no direct interaction with HDAC2.

Mouse brain extracts were incubated with recombinant GST or GST-APPL1 for 3 hr at 4°C. Binding proteins were detected using antibodies against Importin α1 and HDAC2, respectively.

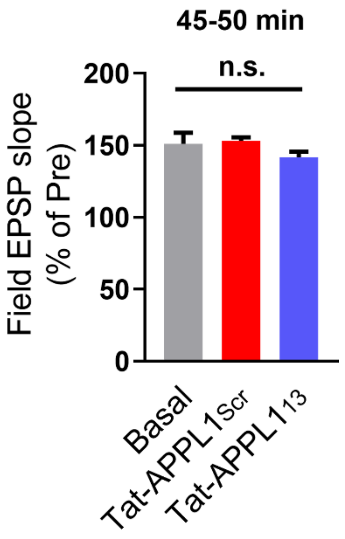

Supplementary Figure 5 Tat-APPL1$_{13}$ has no effect on the early phase of L-LTP.

L-LTP was induced by 4 trains of tetanic stimulation (3 min apart), and perfusion with Tat-APPL1$_{13}$ (n = 8 slices, 8 mice) or with Tat-APPL1$_{Scr}$ (n = 6 slices, 6 mice) has no effect on the initial phase of L-LTP (40-50 min after induction of L-LTP) compared to basal group (n = 7 slices, 7 mice). Data are presented as means ± SEM.

Supplementary References

Qiu, S., Chen, T., Koga, K., et al. (2013). An increase in synaptic NMDA receptors in the insular cortex contributes to neuropathic pain. Sci Signal *6*, ra34.

Qiu, S., Zhang, M., Liu, Y., et al. (2014). GluA1 phosphorylation contributes to postsynaptic amplification of neuropathic pain in the insular cortex. J Neurosci *34*, 13505-13515.

Shechter, D., Dormann, H.L., Allis, C.D., et al. (2007). Extraction, purification and analysis of histones. Nat Protoc *2*, 1445-1457.

Wang, J., Lv, X., Wu, Y., et al. (2018). Postsynaptic RIM1 modulates synaptic function by facilitating membrane delivery of recycling NMDARs in hippocampal neurons. Nat Commun *9*, 2267.
